# Population effect model identifies gene expression predictors of survival outcomes in lung adenocarcinoma for both Caucasian and Asian patients

**DOI:** 10.1371/journal.pone.0175850

**Published:** 2017-04-20

**Authors:** Guoshuai Cai, Feifei Xiao, Chao Cheng, Yafang Li, Christopher I. Amos, Michael L. Whitfield

**Affiliations:** 1 Department of Molecular and Systems Biology, Geisel School of Medicine at Dartmouth, Hanover, New Hampshire, United States of America; 2 Department of Epidemiology and Biostatistics, University of South Carolina, Columbia, South Carolina, United States of America; 3 Department of Biomedical Data Science, Dartmouth College, Hanover, New Hampshire, United States of America; Universitat de Barcelona, SPAIN

## Abstract

**Background:**

We analyzed and integrated transcriptome data from two large studies of lung adenocarcinomas on distinct populations. Our goal was to investigate the variable gene expression alterations between paired tumor-normal tissues and prospectively identify those alterations that can reliably predict lung disease related outcomes across populations.

**Methods:**

We developed a mixed model that combined the paired tumor-normal RNA-seq from two populations. Alterations in gene expression common to both populations were detected and validated in two independent DNA microarray datasets. A 10-gene prognosis signature was developed through a *l*1 penalized regression approach and its prognostic value was evaluated in a third independent microarray cohort.

**Results:**

Deregulation of apoptosis pathways and increased expression of cell cycle pathways were identified in tumors of both Caucasian and Asian lung adenocarcinoma patients. We demonstrate that a 10-gene biomarker panel can predict prognosis of lung adenocarcinoma in both Caucasians and Asians. Compared to low risk groups, high risk groups showed significantly shorter overall survival time (Caucasian patients data: HR = 3.63, p-value = 0.007; Asian patients data: HR = 3.25, p-value = 0.001).

**Conclusions:**

This study uses a statistical framework to detect DEGs between paired tumor and normal tissues that considers variances among patients and ethnicities, which will aid in understanding the common genes and signalling pathways with the largest effect sizes in ethnically diverse cohorts. We propose multifunctional markers for distinguishing tumor from normal tissue and prognosis for both populations studied.

## Introduction

Among different ethnic populations, cancers often present with distinct clinical characteristics in incidence, prevalence, mortality and drug response [1]. The heterogeneity among ethnic groups can be caused by extrinsic environmental factors or intrinsic genetic factors that are population specific. Extrinsic factors such as environment, smoking, or dietary habits, have been shown to contribute to a large proportion of variation in cancer susceptibility [2, 3]. In the past decades, the possible role of intrinsic factors such as genetic variation in cancer heterogeneity is gradually attracting researchers’ attention. For example, smoking-related risk of lung cancer is found to be significantly different among populations, which might be due to the between-ethnic variation in the metabolism of nicotine [4]. Similar discrepancies have been observed with biomarkers. Many molecular biomarkers such as mRNAs, proteins, autoantibodies, microRNAs, and cell-free DNA have been identified as candidate biomarkers for diagnosis and treatment in cancer, but few of them have been validated. Variations in genetic architecture among different ethnic groups make it difficult to validate cancer risk associated SNP markers [5]. In the current study, we hypothesized that integrating data across different populations will identify robust biomarkers by taking between-ethnic genetic variation into account. Here, we focused on the most prevalent lung cancer type, adenocarcinoma. Previous studies have identified lung cancer risk variants including mutations in *EGFR* [6], *HER2* [7], *BRAF* [8] or *KRAS* [9], and gene fusions of *RET*, *ALK or ROS1* [10]. However, information on gene expression in a tumor adds biological context to lung cancer prognosis by identifying differentially expressed genes and inferring pathway activation. We analysed datasets from two cohort studies for Caucasians and Asians, which were generated by RNA sequencing (RNA-seq) [11].

In studies of cancer, comparing paired tumor and anatomically matched-adjacent normal tissues is an effective approach to alleviate the bias from patient variations as well as systematic error. In our study, we analyzed RNA-seq data from 58 primary solid tumors and anatomic-site matched normal tissue pairs from The Cancer Genome Atlas (TCGA) with patients self-identified as Caucasian [12]. Another dataset analyzed 77 primary solid tumor and anatomic-site matched normal tissue pairs from lung adenocarcinoma patients with Korean and East Asian descent [13]. By investigating gene expression patterns in these two populations, we found heterogeneous expression changes in Caucasians and Asians. Considering both population-specific and patient-specific genetic architectures, a mixed model was proposed to identify the candidate biomarkers adjusting tissue, ethnicity, as well as other latent confounding factors in the two cohorts. As a result, a set of consistent differentially expressed genes (DEGs) in Caucasians and Asians was identified. Using those cohort-common DEGs as possible candidates for predicting survival outcomes, we also selected a panel of transcriptome markers for lung adenocarcinoma prognosis for both Caucasian and Asian patients.

## Methods

### Datasets and pre-processing procedures

Two RNA-seq and three DNA microarray datasets were used in this study (Table 1), including the following:

**Table 1:** Datasets used.

Platform	Name	Population	Paired	Survival data available	Accession
**RNA-seq**	Caucasian-seq	Caucasian	Yes	Yes	TCGA[12]
	Asian-seq	Asian	Yes		GSE40419[13]
**Microarray**	Caucasian-array	Caucasian	Yes		GSE19804[14]
	Asian-array	Asian	Yes		GSE10072[15]
	GSE8894	Asian		Yes	GSE8894[16]

#### Caucasian RNA-seq study

We downloaded an Illumina HiSeq 3.1.12.0 lung adenocarcinoma RNA-seq dataset from the TCGA database [12]. The dataset contains 457 tumor and 58 paired normal tissues, in which 53 pairs were from Caucasian patients. 2x48 bp pair-end RNA-seq reads were aligned to the Ensembl GRCh37 human reference genome by MapSplice [17]. Read counts of each gene were then estimated by RSEM [18]. The overall survival data and clinical variables of the patients were also downloaded from TCGA.

#### Asian RNA-seq study

Tumor and normal paired RNA-seq data of 77 lung adenocarcinoma patients were downloaded from Gene Expression Omnibus (GEO) with the accession number GSE40419 [13]. 100-bp pair-end reads were generated from Hiseq sequencing platform. We used Tophat to align reads to the Ensembl GRCh37 human reference genome and HTSeq to calculate the counts mapped to each gene [19, 20]. Clinical information of the patients was downloaded from the public website (http://genome.cshlp.org/content/22/11/2109/suppl/DC1).

#### Three microarray studies

We also used three microarray datasets for validation, which were available in NCBI GEO database under accession numbers GSE19804, GSE10072 and GSE8894. All three mRNA expression DNA microarray-derived datasets were generated with Affymetrix GeneChip Human Genome U133 Arrays. Both GSE19804 and GSE10072 studied tumor and paired normal tissues. 60 Asian patients in Taiwan were enrolled in the GSE19804 study [14] and gene expression data from 33 Caucasian patients were available in the GSE10072 study [15]. Gene expression raw signals from GSE19804 and GSE10072 studies were processed and normalized using the robust multiarray average (RMA) expression measure method [21]. We used the GSE8894 dataset to validate selected prognosis markers, in which transcriptome expression and recurrence-free survival information of 62 Asian patients were available [16]. We downloaded GCRMA normalized data of all probe sets from the GSE8894 study. For genes with multiple probes, the probe with the maximum average expression values in all samples was selected to represent the gene expression.

### Mixed effect models

Reads per kilobase per million mapped reads (RPKM) values were calculated from RNA-seq gene counts, and were *log*2 transformed to improve normality. Then we imputed missing values using the K-nearest neighbor method [22].

Because of the dependency of measurements within a patient from a specific population, for each gene, we applied a mixed effect linear model,

$$logit\left(\text{Pr}\left(y_{i}=1\right)\right)=x_{i}^{T}\beta +z_{i}^{T}\gamma +q_{i}^{T}\delta +\varepsilon$$ (1)

to detect tumor-normal DEGs. Here, *y*_*i*_ is the dichotomous outcome for the *i*-th patient which is 1 for tumor tissue and 0 for normal tissue. For the *i*-th patient, *x*_*i*_ indicates a vector of variables representing the *log*2 scaled RPKM values for a set of *p* genes (*x*_*i*1_,*x*_*i*2_,*x*_*i*3_,…,*x*_*ip*_)^*T*^.*β* = (*β*_1_,*β*_2_,*β*_3_,…,*β*_p_)^*T*^ is a vector of regression coefficients to be estimated. When detecting DEGs, we performed a gene-wise testing in which *p* = 1. *z*_*i*_ and *q*_*i*_ are indexes of patient ID and populations (Caucasians and Asians), respectively, which account for subject-specific effects. *γ* and *δ* are unknown vectors of random effect regression coefficients that model the effects from populations and individuals, respectively, whereas *E*(*γ*) = 0 and *E*(*δ*) = 0. The restricted maximum likelihood (REML) method was used to estimate parameters *β*, *γ* and *δ*. F-test was performed to test the association of gene expression with the disease outcome in the full model (Eq 1) comparing to the following null model without gene expression variables (Eq 2) as

$$logit\left(\text{Pr}\left(y_{i}=1\right)\right)=z_{i}^{T}\gamma +q_{i}^{T}\delta +\varepsilon$$ (2)

*p*-values were adjusted to control the multiple testing false discovery rate using the Benjamini-Hochberg method.

We also tested the DEGs in each specific population with the full and null models shown in Eqs 3 and 4, in which the population effect *q*_*i*_ was null within one specific population,

$$logit\left(\text{Pr}\left(y_{i}=1\right)\right)=x_{i}^{T}\beta +z_{i}^{T}\gamma +\varepsilon$$ (3)

$$logit\left(\text{Pr}\left(y_{i}=1\right)\right)=z_{i}^{T}\gamma +\varepsilon$$ (4)

### Biomarker selection

Logistic regression and Cox proportional hazard model were used for tumor-normal classification and prognosis prediction separately. The *l*1 penalized regression technique LASSO was used to select predictive genes as potential biomarkers [23]. For the *i*- th patient, we still use the vector *x*_*i*_ to denote the expression of a set of biomarker candidates, and *h*_*i*_ to denote the log odds of cancer outcome or log hazard ratio of death. The regression coefficients *βs* were estimated with the *l*1 penalized term *λ*||*β*||_2_ according to Eq 5 as

$$\hat{\beta }=argm{\text{ax }}_{\beta }\left[∥x_{i}^{T}\beta -h_{i}{∥}_{2}-\lambda ∥\beta {∥}_{2}\right]$$ (5)

where *λ* was a tuning factor which was determined by minimizing the deviance.

Variables with $\hat{\beta }$ larger than 0 were considered as potential predictive biomarkers. To evaluate the goodness of model fitting, we used the 5 fold cross validation strategy by randomly splitting data into a training dataset with 80% of the sample and a test dataset with the rest for 5 times. The coefficient of determination *R*^*2*^ was calculated as *$1-\frac{RSS}{TSS}$*, where *RSS* was the residual sum of squares and *TSS* was the total sum of squares.

### Clustering, enrichment and association testing

To evaluate the genetic distance among samples in Asian and Caucasian, hierarchical clustering was applied based on the expression profiling of identified DEGs from RNA-seq. The IPA software (http://www.ingenuity.com/products/ipa) was used to identify gene set enriched signaling pathways, upstream regulators and their target networks. Also, we applied linear regression, logistic regression and ordinal data analysis to investigate the association between the risk score and clinical features. All data manipulations, statistical analyses and visualizations were accomplished using R 3.0.2.

## Results

### Cohort-common Differential Expression Genes (DEGs)

First, we investigated differential expression between tumor and paired normal tissues from two ethnically different cohorts of patients with lung adenocarcinoma. The tumor-normal log ratios of gene expression were consistent across Asian and Caucasian RNA-seq studies with cohort specific variations (S1A Fig). 4418 genes had significant differential tumor-normal log ratios of gene expression between populations; FDR adjusted *p*-values showed an overabundance of small values rather than being uniformly distributed (S1B Fig).

**Fig 1:**
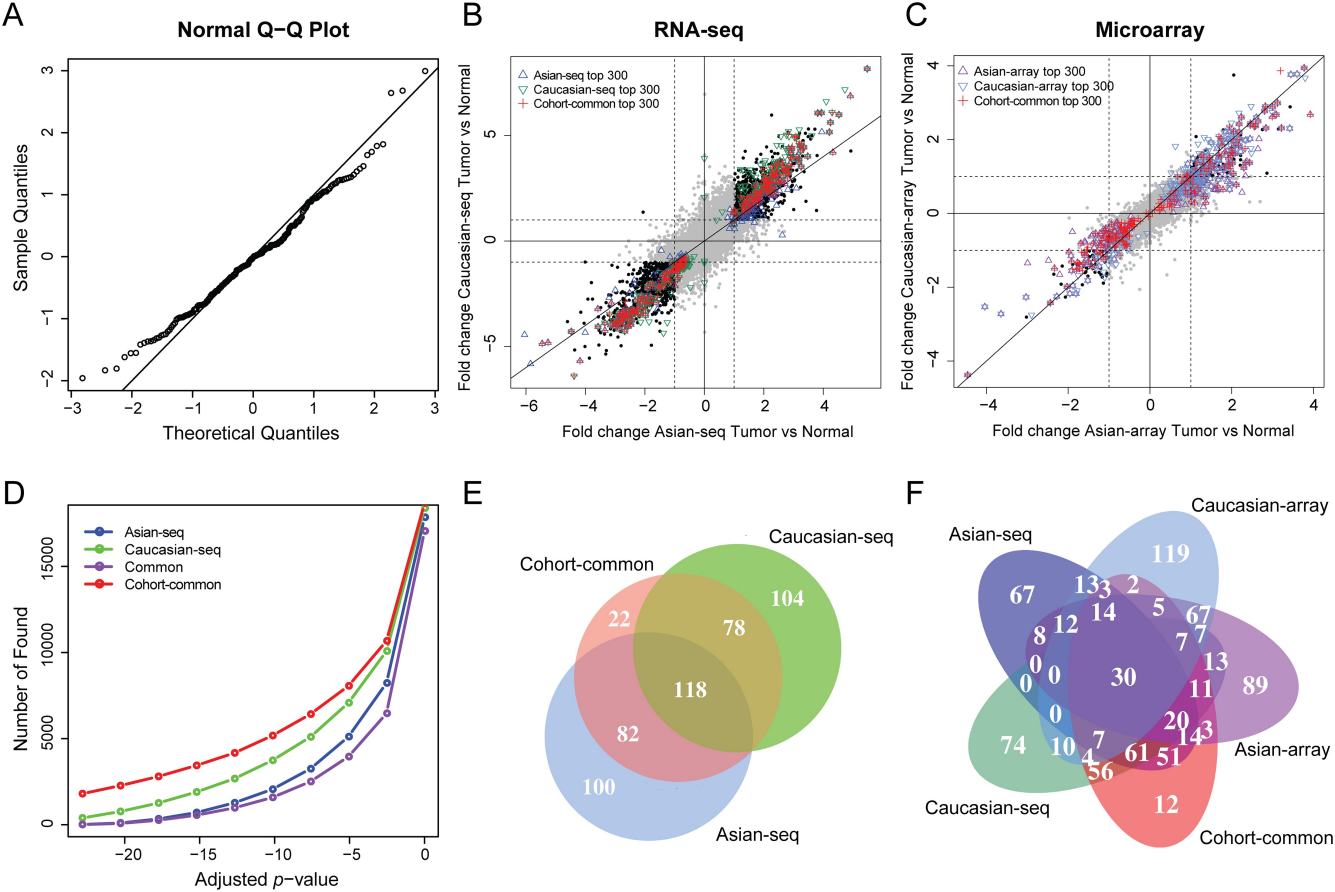
Cohort-common and cohort-specific detections of DEGs. (A) Q-Q plot of residuals of one randomly selected gene from 4418 genes having significant differential tumor-normal log ratios of gene expression between populations. (B) Comparison of detections from Asian and Caucasian RNA-seq studies. (C) Comparison of detections from Asian and Caucasian microarray studies. (D) Comparison of discovery rates from population-common and population-specific analyses. (E) Venn diagram of the top 300 DEGs from Asian and Caucasian RNA-seq studies. (F) Venn diagram of the top 300 DEGs from all RNA-seq and microarray studies.

To identify consistent DEGs in both Caucasian and Asian cohorts, we designed a mixed effect model with normally distributed residuals (Fig 1A). Cohort-common DEGs were highly regulated in tumor tissues in both Asian and Caucasian studies (Fig 1B), which were also found consistently to be highly differentially expressed in independent DNA microarray studies (Fig 1C). We compared the top 300 DEGs from population-specific (Asian-seq, Caucasian-seq) and population-common analyses in Fig 1E (summary of the top 300 DEGs were shown in S1–S3 Tables). All 118 DEGs identified in both Asian and Caucasian cohorts were detected by the cohort-common analysis as well. Comparing the cohort-common genes from the RNA-seq datasets and four population-specific gene sets from RNA-seq (Asian-seq, Caucasian-seq) and microarray (Asian-array, Caucasian-array) datasets, we found a robust differential expression of the cohort-common genes (Fig 1F). As expected, the cohort-common analysis showed greater power of detection because of the increased sample size, which identified more DEGs than the cohort-specific analyses at the same significance thresholds (Fig 1D). Interestingly, we also observed that Caucasian-seq analysis detected more DEGs than Asian-seq analysis (Fig 1D), which was probably due to the larger fold changes (Fig 1B).

**Fig 2:**
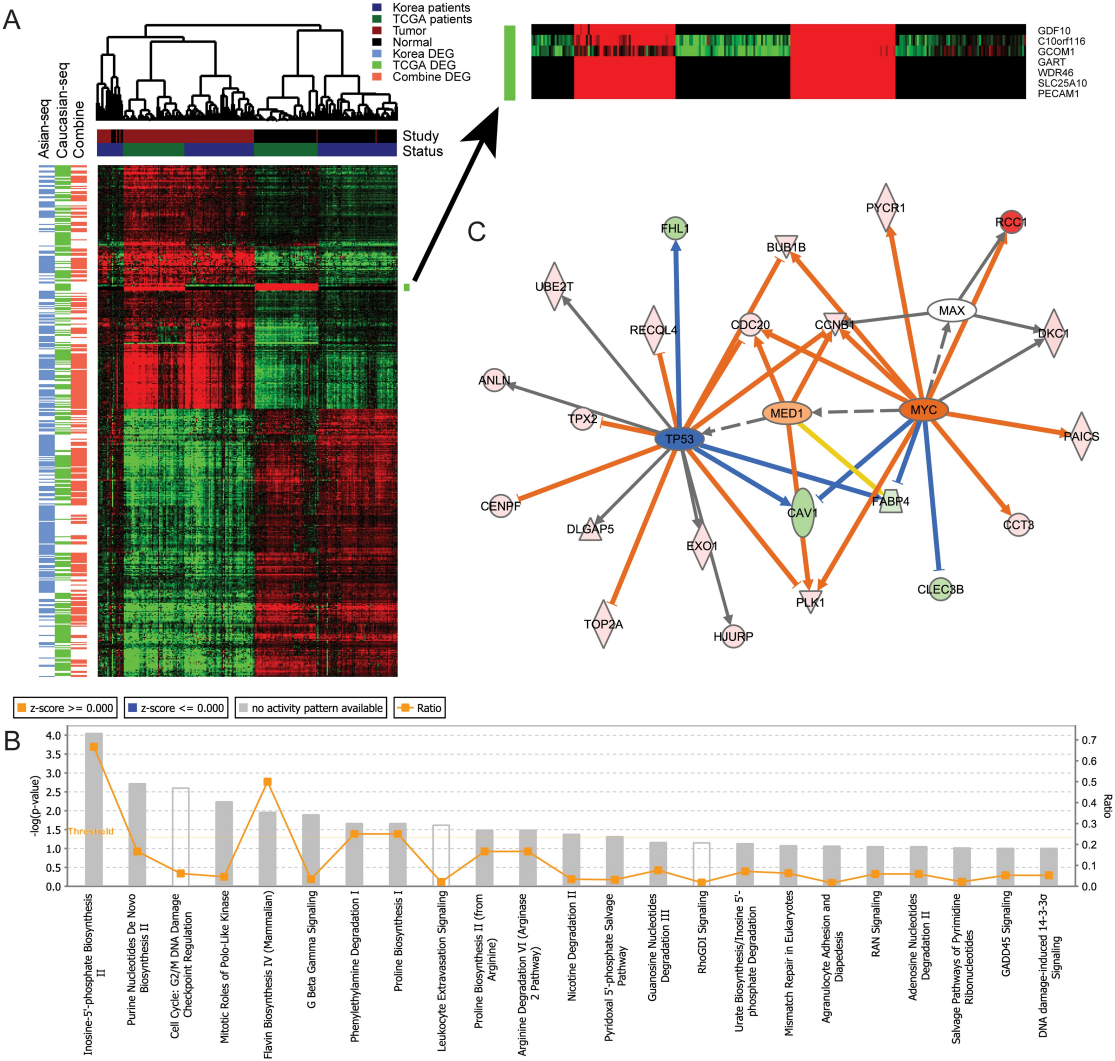
Enrichment analyses of cohort-common DEGs. (A) Hierarchical clustering of gene expression of cohort-common and cohort-specific DEGs in both Asian and Caucasian RNA-seq studies. (B) 118 cohort-common DEGs enriched pathways. (C) Upstream regulators and their target networks enriched in 118 cohort-common DEGs.

Hierarchical clustering of all RNA-seq cohort-common and cohort-specific DEGs showed that the expression profiling of the top tumor-normal DEGs in Caucasians and Asians were highly consistent (Fig 2A). However, the expression for several genes, such as *GDF10*, *C10orf116*, *GCOM1*, *GART*, *WDR46*, *SLC25A10* and *PECAM1* were population specific, in which *C10orf116* [24], *GART* [25] and *SLC25A10* [26] had been reported to be functional in metabolic processes. These results were consistent with the previous findings that Asians and Caucasians had significantly different metabolic profiles [27]. Interestingly, several tumor samples in the Asian cohort showed a similar expression pattern with normal samples. These “normal-like” samples might account for the smaller tumor-normal log ratios in this cohort shown in Fig 1B.

The 118 common DEGs were enriched in pathways related to cell proliferation, such as cell cycle and biosynthesis of compounds including inosine-5’-phosphate, purine nucleotides, flavin etc (Fig 2B). Although *TP53* showed increased expression (*p*-value = 1.55 × 10^−11^) and *MYC* was slightly decreased (*p*-value = 0.002) in lung cancer tumors compared to paired normal tissues, *TP53* target genes showed decreased expression (Fig 2C), consistent with the frequent observation of mutations of TP53 in adenocarcinomas [28]. Furthermore, *MYC* target genes showed increased expression indicating potential MYC pathway activation.

### A 10-marker panel for lung adenocarcinoma prognosis prediction

**Fig 3:**
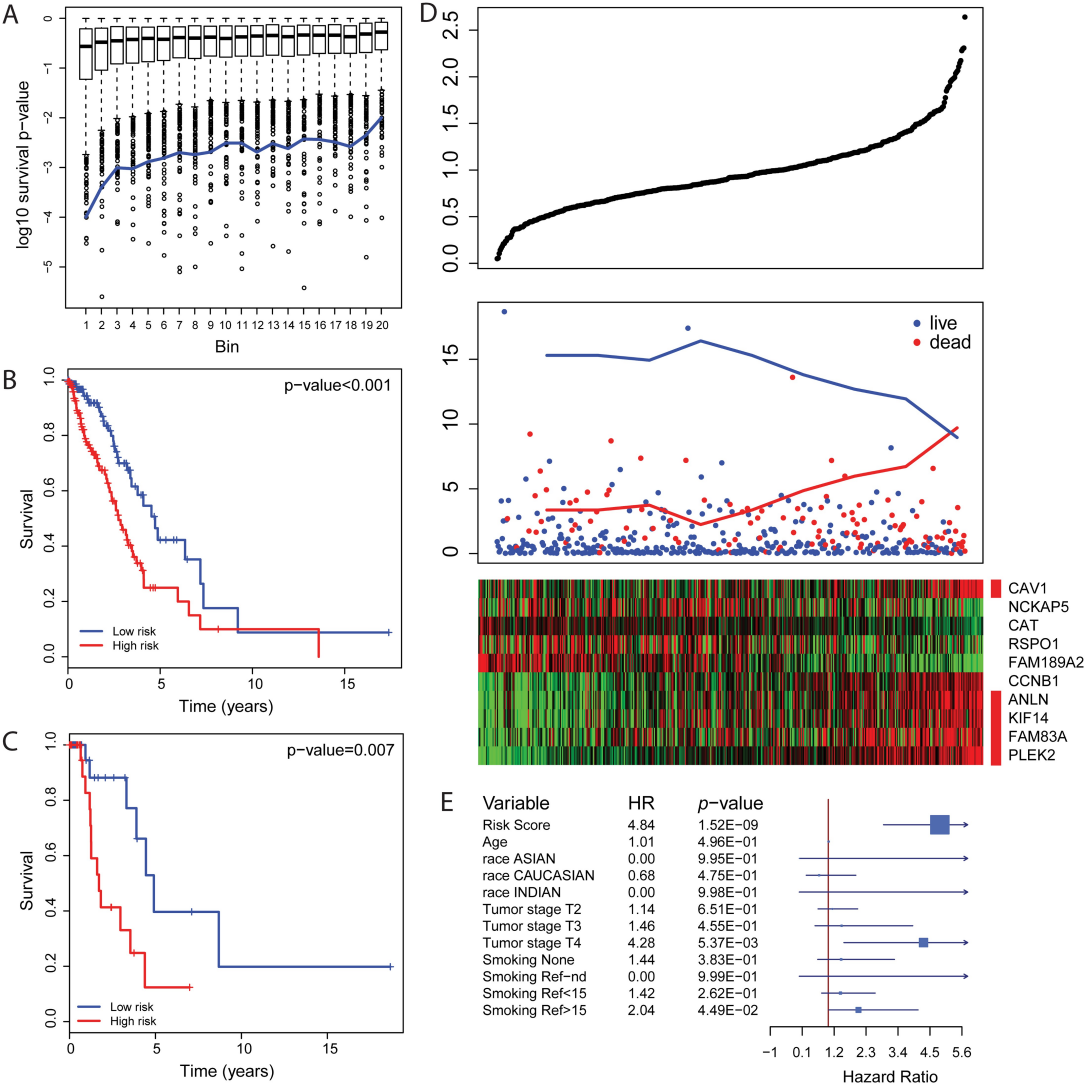
Prognosis markers. (A) Tumor-normal DEGs have more power to predict survival outcome. Genes were ranked according to the significance of differential expression between tumor and normal tissues and were categorized into 20 bins evenly. The blue line indicates the rescaled discovery rate of survival associated genes. (B, C) Kaplan-Meier plot of high-risk and low-risk groups from training and test datasets. (D) From top to bottom, (panel 1) predicted risk scores; (panel 2) survival records of patients; (panel 3) gene expression of the 10 selected markers. Structural arrangement controlling genes are indicated by red bars. X axis in all 3 panels shows the patients in the same order. (E) Statistics of multiple regression with risk score and other clinical features as co-factors.

We tested the identified cohort-common DEGs for predicting tumor prognosis using the TCGA dataset. Generally, tumor-normal DEGs had more power to predict prognosis than non-DEGs (Fig 3A). 30 out of 118 cohort-common DEGs were significantly associated with the risk of death with FDR adjusted *p*-values less than 0.05 (coefficients and significances were shown in S2A Fig and c-indexes were shown in S2B Fig). From those 30 genes, 10 prognosis markers were selected using LASSO with *λ* = 10^−3^ (S2C and S2D Fig), including five cell structural arrangement related genes (*CAV1*, *FAM83A*, *PLEK2*, *KIF14* and *ANLN)*, two cell cycle and growth related genes (*CCNB1* and *RSPO1*), an antioxidant gene *CAT* and two function-unknown genes *FAM189A2* and *NCKAP5*. Risk scores were calculated as *CAV1* * 0.12 + *FAM83A* * 0.017 + *ANLN* * 0.01 + *PLEK2* * 0.17 + *KIF14* * 0.043 + *CCNB1* * 0.015—*RSPO1* * 0.0048—*FAM189A2* * 0.091—*NCKAP5* * 0.022—*CAT* * 0.036. With the mean of risk scores as the threshold, we assigned patients into high risk and low risk groups. The high risk group had significantly shorter survival time than the low risk group in both training (hazard ratio = 2.12, *p*-value = 6.58 × 10^−4^, Fig 3B) and test datasets (hazard ratio = 3.63, *p*-value = 0.007, Fig 3C), which were randomly split from the TCGA dataset. The risk scores showed statistical significance for patient prognosis for tumor stage I/II lung adenocarcinoma (hazard ratio = 2.51, *p*-value = 4.19 × 10^−5^, S3 Fig Left). For tumor stage III/IV lung adenocarcinoma, high risk patients had 2.46 times higher hazard risk than low risk patients, which was not statistical significance (*p* = 0.075) due to the limited number of late stage patients (S3 Fig Right). Consistent with these results, patients with higher risk scores had a higher likelihood of death (Fig 3D Top). As expected, the 10 selected markers showed significant alterations in gene expression corresponding to the increased risk score and death proportion (Fig 3D). Consistently, each of these 10 gene markers showed significant power in discriminating patients with low risk and high risk of death (S4 Fig). *CAV1* and *NCKAP5* showed the weakest prognostic power individually but contribute to prognosis in the combined risk score.

### Prognostic power with clinical survival risk co-factors

**Fig 4:**
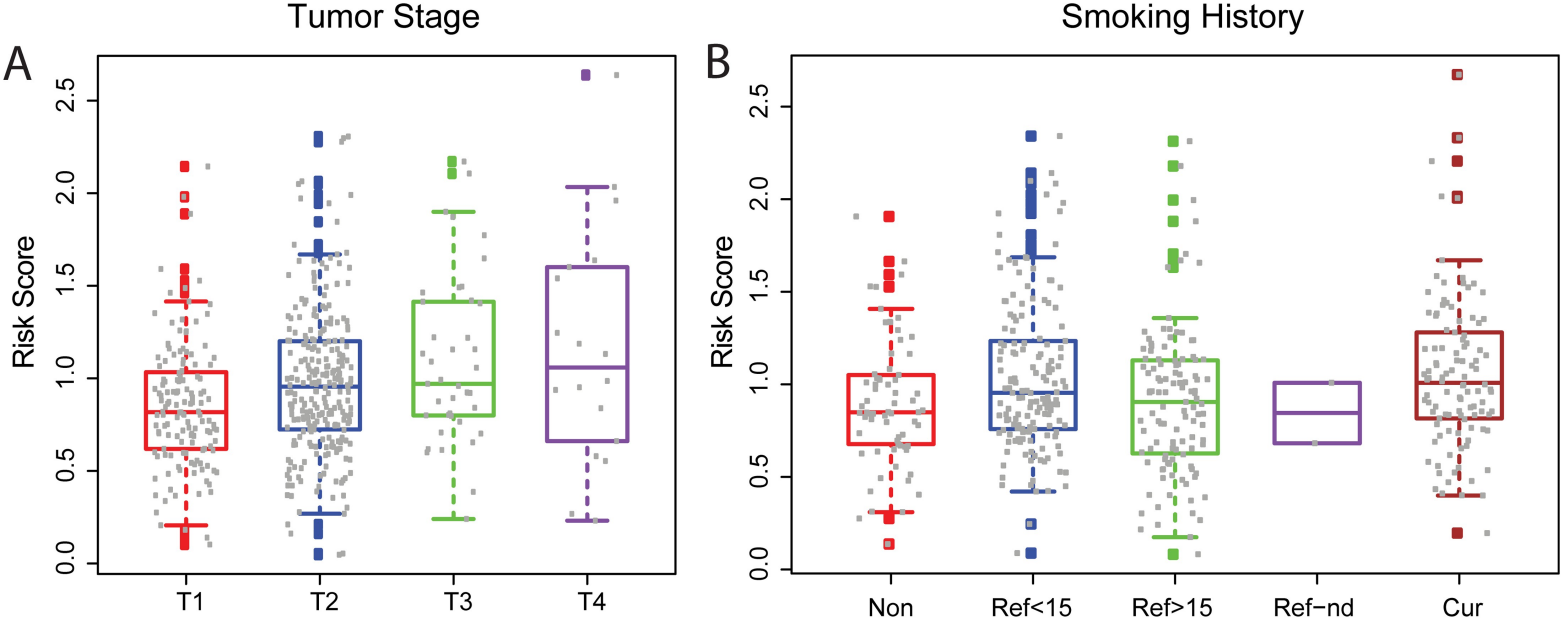
Association study of survival risk score with clinical features. (A) AJCC Tumor stage. (B) Smoking history, Non: Lifelong Non-smoker; Ref<15: Current reformed smoker for < or = 15 years; Ref>15: Current reformed smoker for > 15 years; Ref-nd: Current Reformed Smoker, Duration Not Specified; Cur: Current smoker.

We investigated the association of the 10-marker prognostic risk score with clinical features including age, race, disease outcome, American Joint Committee on Cancer (AJCC) TNM tumor staging information (cancer metastasis stage, neoplasm disease lymph node stage and tumor stage), lung capacity indicators (FEV1, FEV1/FVC, DLCO) and smoking history. In Fig 4, we found that the risk score was significantly positively correlated with tumor stage. Also as expected, current smokers had the highest risk scores, non-smokers had the lowest risk scores and former smokers who have quit for longer durations had lower scores compared to more recent quitters. A positive correlation with neoplasm disease lymph node stage, and a negative correlation with weak lung capacity (low FEV1 and DLCO) were also observed (S5 Fig). However, we observed that associations between risk score and age, race, cancer metastasis stage or FEV1/FCV percentage were not significant. The summary of statistics from association tests is shown in S4 Table. To evaluate the predictive power of the risk score with those clinical co-factors including age, race, tumor stage and smoking history, we performed a multiple regression analysis and found that risk score dominated the prediction with weak additional contributions from tumor stage and smoking history (Fig 3E).

### Selected markers were applicable for both prognosis and discrimination for both Asian and Caucasian populations

**Fig 5:**
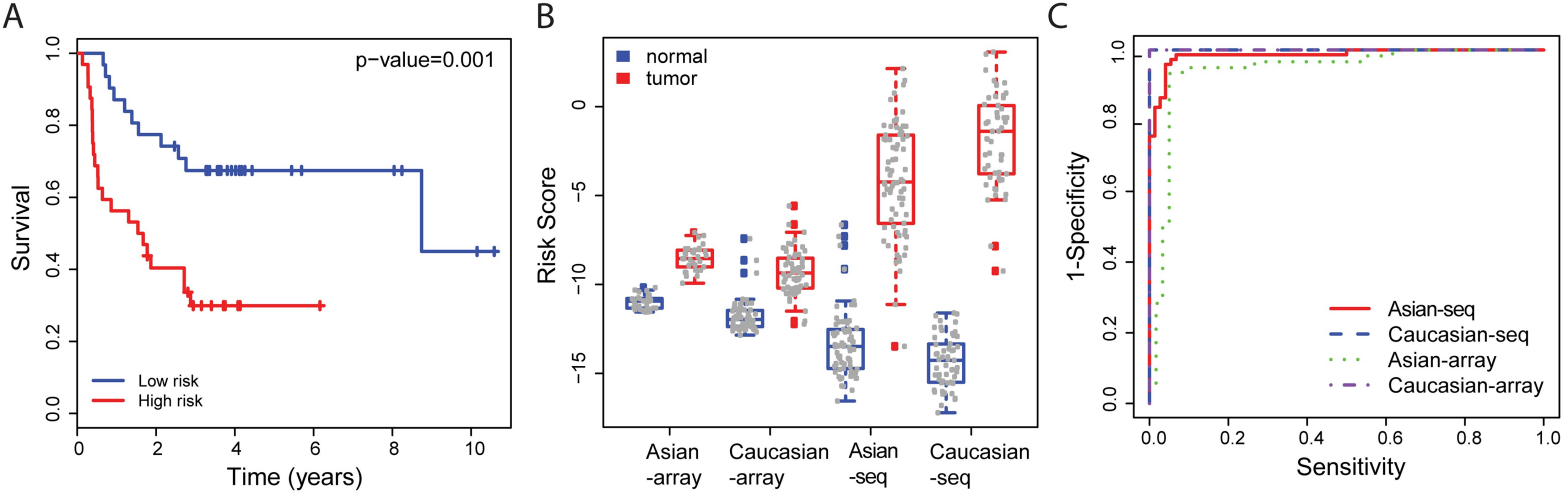
Power of prognosis markers. (A) Kaplan-Meier plots of high-risk and low-risk patients grouped by prognosis markers on validate dataset GSE8894. (B) Prognosis risk scores in all RNA-seq and microarray datasets. (C) ROC curves for tumor-normal discrimination by selected markers in all RNA-seq and microarray datasets.

Above, we selected prognosis markers from the cohort-common DEGs with the expectation that they have high predictive power in both populations. Here, we evaluate the performance of the prediction model using this marker panel in an Asian microarray study (GSE8894). This evaluation showed significant difference between the high-risk and low-risk groups (hazard ratio = 3.25, *p*-value = 0.001) (Fig 5A). In contrast, 10 prognosis markers selected from the 104 Caucasian specific DEGs showed in Fig 1E presented less ability to discriminate high-risk group from low-risk group (hazard ratio = 2.20, *p*-value = 0.028, figure not shown).

Moreover, the selected prognosis markers had high power to differentiate tumor from normal tissue. A large difference of prognosis risk score was found between cancer and healthy control tissues from all RNA-seq and microarray studies (Fig 5B). A logistic model was trained for discriminating tumor from normal tissue, by which we obtained high power of discrimination showed in the ROC curve (Fig 5C).

## Discussion

Our results demonstrate that although Asian and Caucasian studies produced consistent genome-wide expression profiles, many genes have distinct tumor-normal alterations between these two specific populations. Therefore, we designed a mixed effect model to include random effects from ethnicity and patient subject for detecting the cohort-common DEGs. We selected 10 genes as biomarkers of prognosis from the cohort-common DEGs and found powerful discrimination of tumor and normal tissues in both Asian and Caucasian populations.

The differences between Asian and Caucasian cohort studies were captured and modeled in our mixed effect model. Subject-average effects were estimated as well as subject-specific effects from each subject, ethnicity and other confounding factors between studies. Thus, we were able to characterize subject-specific confounders from individuals and populations, and capture the true phenotype-genotype associations. Applying it, we detected 118 cohort-common DEGs and found that *TP53* and *MYC* targeted genes were abnormally regulated in tumor tissues. This indicates that for lung adenocarcinoma patients from both Asian and Caucasian cohort studies, the apoptosis pathway might be turned down and cell-cycle pathway might be powered up. Also, we observed that metabolic genes including *C10orf116* [24], *GART* [25] and *SLC25A10* were differentially expressed between Caucasian and Asian populations. However, additional studies are required to validate that these discrepancies are population-specific and not due to technical variation.

This study demonstrated the relevance of tumor-normal discrimination and prognosis prediction in two populations using the same gene expression markers. Our findings provide the logical basis for finding universal makers for both tumor discrimination and prognosis for cost and time effectiveness. Also, our mixed effect model detected cohort-common DEGs to provide a maker candidate pool for both Asian and Caucasian populations. With these logical basis and candidate pool, we selected 10 markers and validated their capability for tumor discrimination and prognosis of lung adenocarcinoma in both Asian and Caucasian specific studies with a high predictive power. Of the ten genes selected as biomarkers, five (*CAV1*, *FAM83A*, *PLEK2*, *KIF14* and *ANLN*) are associated with key events in cell division including signal transduction, the actin cytoskeleton or in microtubule dynamics. All five genes were positively associated with survival time, consistent with previous reports that these genes are oncogenic in lung and other cancers [29–33]. The 10-gene biomarker panel also includes *CCNB1*, which is a key cell-cycle regulator and known prognostic predictor of lung adenocarcinoma [34]. The antioxidant gene *CAT* was found to be negatively associated with the risk of death. This is consistent with that the overexpression of *CAT* leads to a less aggressive phenotype of cancer cells [35, 36]. Despites several gene expression candidate marker sets have been proposed [37–39], they are from single population studies and lack of reproducibility for clinical application. In the current study, we selected prognosis markers from the whole transcriptome RNA-seq quantification, aiming to achieve a higher prognosis power than previous microarray studies or PCR studies of empirically selection markers. Furthermore, we considered the population genetics variations into our analysis thus our makers have higher potential in application across populations compared to previous studies developed based on single populations.

In clinical practice, tumor stage is the main prognostic indicator for treating lung adenocarcinoma. Surgical resection is the standard treatment for tumor stage I/II patients, whereas chemotherapy and radiation are suggested to treat tumor stage III/IV patients. The 10-gene marker panel demonstrated statistical significance for patient prognostication, particularly for early stage lung adenocarcinoma, suggesting surgery may be insufficient for the high-risk early patients and may be improved with additional adjuvant chemotherapy.

Prognostic risk scores from the 10-gene biomarker panel were significantly correlated with known clinical survival risk factors including tumor stage, FEV1 and DLCO, and smoking history. However, this new panel showed the highest prognosis power in multivariate analysis. Further in the future, other potential predictors will included in the model, such as mutations of *EGFR*, *HER2*, *BRAF* or *KRAS*, fusions of *RET*, *ALK* or *ROS1* and others.

In conclusion, this study uses a statistical framework to detect DEGs between tumor and normal tissues that considers variances among patients and ethnicities, as well as confounding factors such as microarray or RNA-seq platform and data processing strategies. Such a method can help us understand the genes and signalling pathways with the largest effect sizes in ethnically diverse cohorts. We propose multifunctional markers for distinguishing tumor from normal tissue and prognosis for both populations studied. This study provides a strategy for identifying biomarkers from high-throughput transcriptome profiling data across cohorts of diverse patients.

## Supporting information

S1 Fig Comparison of tumor-normal gene expression alterations between Asian and Caucasian populations. (A) Comparison of tumor-normal log ratios from Asian and Caucasian RNA-seq studies. (B) Distribution of *p*-values from differential testing on tumor-normal log ratios.
(TIF)

S2 Fig Selection of prognosis markers. (A) Univariate survival analysis statistics of genes with FDR less than 0.05. (B) c-indexes of genes with FDR less than 0.05. (C) Cross-validated deviance of LASSO fit. (D) Prediction statistics of selected markers.
(TIF)

S3 Fig Prognostic power of risk score in patients within early and late stages. Left: Kaplan-Meier plot of high risk and low risk groups of tumor stage I/II patients. Right: Kaplan-Meier plot of high risk and low risk groups of tumor stage III/IV patients.
(TIF)

S4 Fig Kaplan-Meier plots of each individual prognosis marker. Patients were grouped based on the mean of gene expression value of each individual prognosis marker.
(TIF)

S5 Fig Association study of survival risk score with clinical features. (A) AJCC Neoplasm disease lymph node stage. (B) Pre-bronchodilator FEV1. (C) Post-bronchodilator FEV1. (D) Diffusing capacity of the lungs for carbon monoxide (DLCO).
(TIF)

S1 Table The top 300 DEGs from Asian-seq analyses. (XLSX)

S2 Table The top 300 DEGs from Caucasian-seq analyses. (XLSX)

S3 Table The top 300 DEGs from population-common analyses. (XLSX)

S4 Table The summary of statistics from association tests on 10-marker prognosis risk score and clinical features. (XLSX)
