# Supplementary figures and images for: Population effect model identifies gene expression predictors of survival outcomes in lung adenocarcinoma for both Caucasian and Asian patients
Source: PLoS One. 2017 Apr 20;12(4):e0175850. doi: 10.1371/journal.pone.0175850 (PMC5398559; doi:10.1371/journal.pone.0175850)

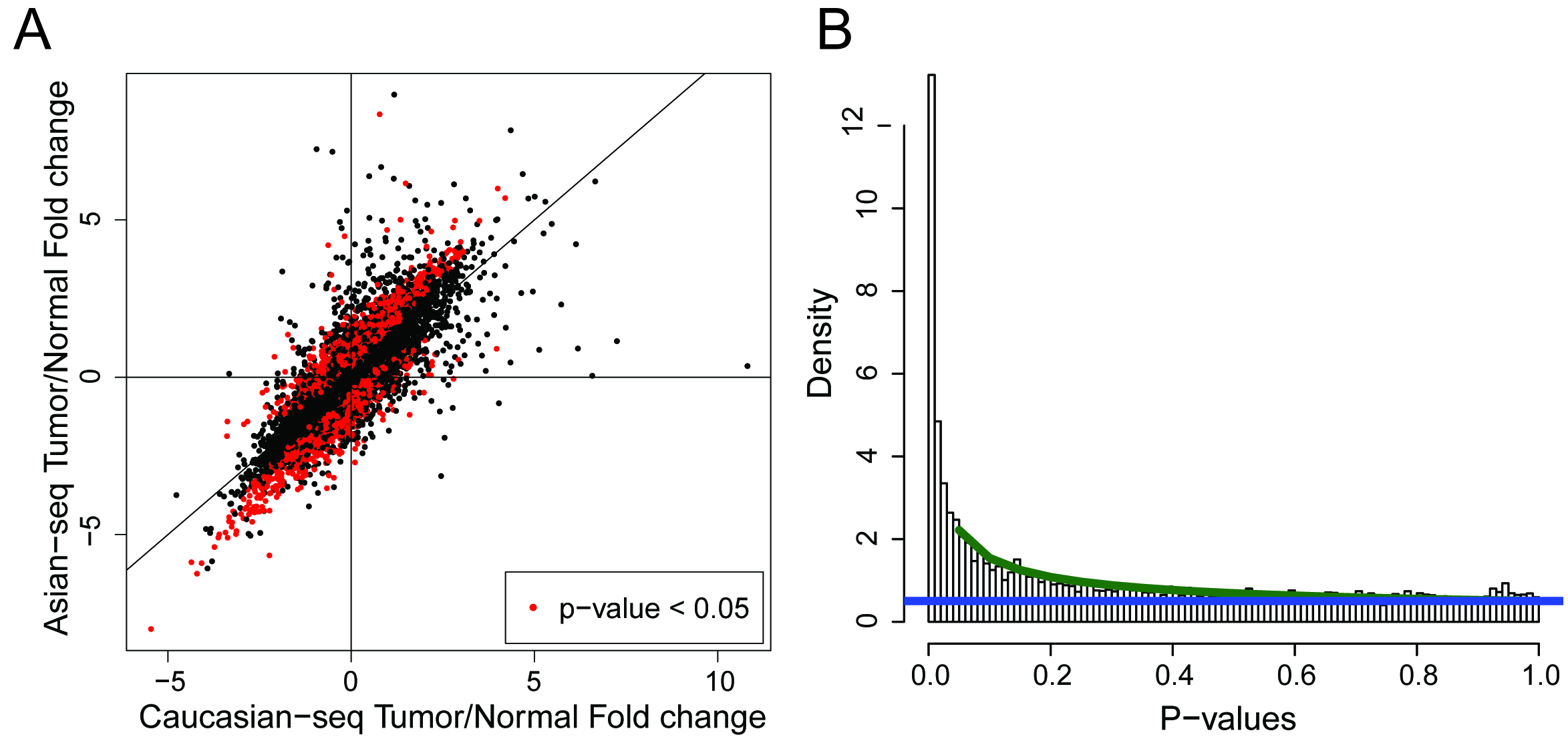

Supplement: S1 Fig — (A) Comparison of tumor-normal log ratios from Asian and Caucasian RNA-seq studies. (B) Distribution of p-values from differential testing on tumor-normal log ratios. (TIF) [file pone.0175850.s001.tif]

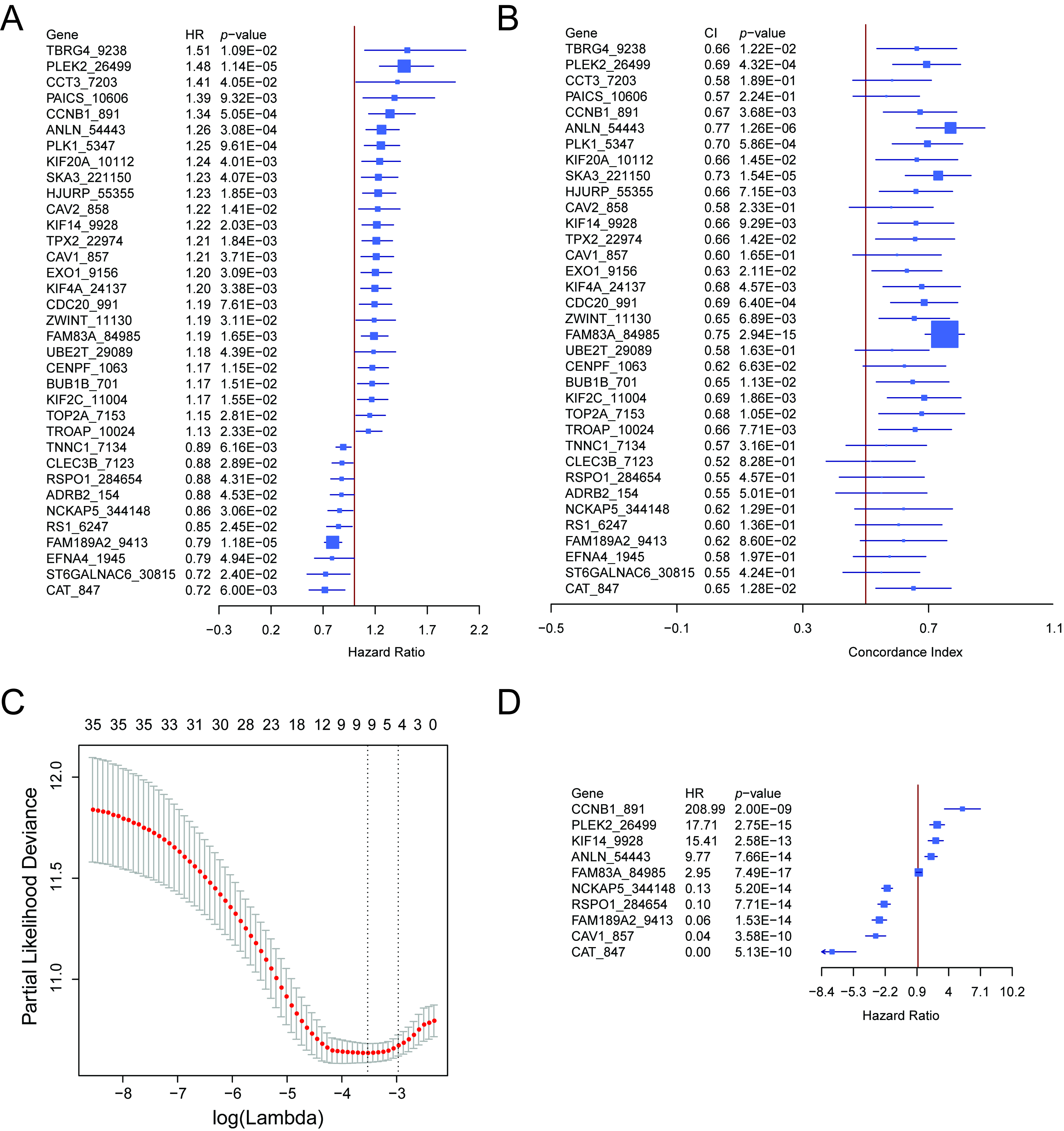

Supplement: S2 Fig — (A) Univariate survival analysis statistics of genes with FDR less than 0.05. (B) c-indexes of genes with FDR less than 0.05. (C) Cross-validated deviance of LASSO fit. (D) Prediction statistics of selected markers. (TIF) [file pone.0175850.s002.tif]

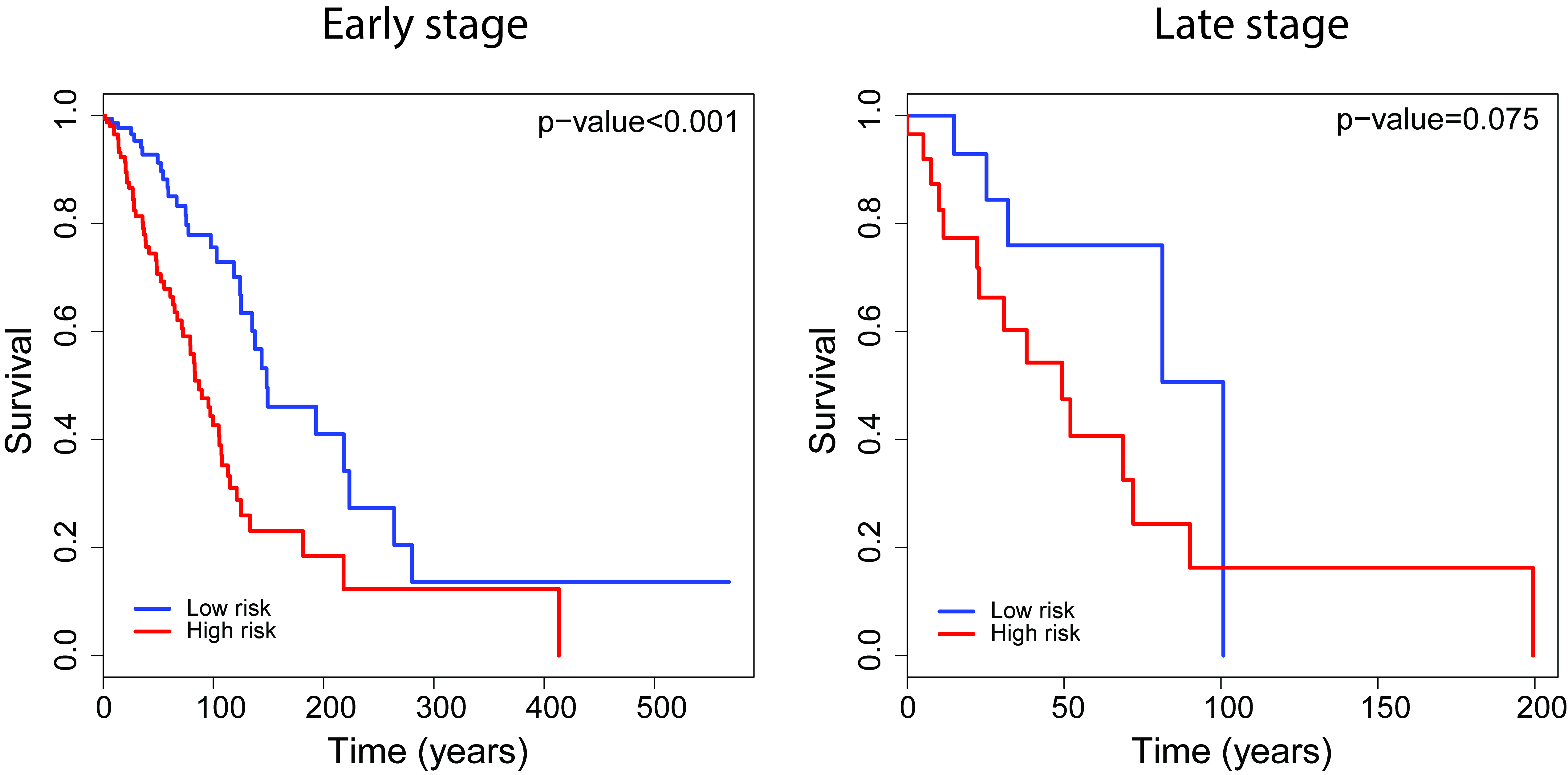

Supplement: S3 Fig — Left: Kaplan-Meier plot of high risk and low risk groups of tumor stage I/II patients. Right: Kaplan-Meier plot of high risk and low risk groups of tumor stage III/IV patients. (TIF) [file pone.0175850.s003.tif]

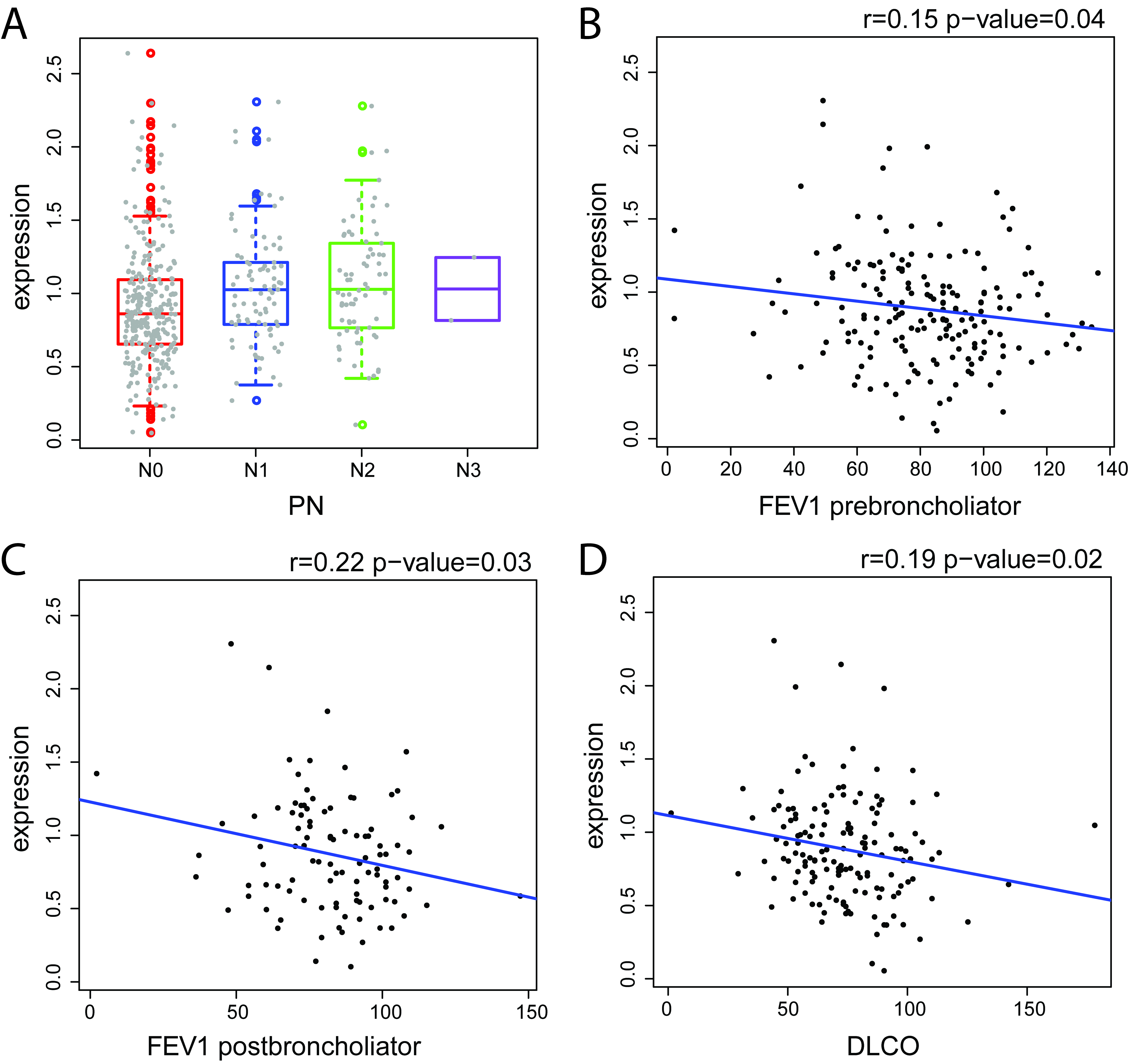

Supplement: S5 Fig — (A) AJCC Neoplasm disease lymph node stage. (B) Pre-bronchodilator FEV1. (C) Post-bronchodilator FEV1. (D) Diffusing capacity of the lungs for carbon monoxide (DLCO). (TIF) [file pone.0175850.s005.tif]
